# Plant Essential Oils as Biopesticides: Applications, Mechanisms, Innovations, and Constraints

**DOI:** 10.3390/plants12162916

**Published:** 2023-08-10

**Authors:** Ipsa Gupta, Rishikesh Singh, Suganthi Muthusamy, Mansi Sharma, Kamaljit Grewal, Harminder Pal Singh, Daizy R. Batish

**Affiliations:** 1 Department of Botany, Faculty of Science, Panjab University, Chandigarh 160014, India; ipsagupta1996@gmail.com (I.G.); rishikesh.iesd@gmail.com (R.S.); 2 Department of Biotechnology, Vels Institute of Science, Technology & Advanced Studies, Pallavaram, Chennai 600117, India; suganthi.sls@velsuniv.ac.in; 3 Department of Environment Studies, Faculty of Science, Panjab University, Chandigarh 160014, India; sharma.m2409@gmail.com; 4 Department of Botany, Khalsa College for Women, Civil Lines, Ludhiana 141001, India; kamalkcw@gmail.com

**Keywords:** agricultural sustainability, botanical pesticides, encapsulation, nanoformulations, plant extracts, weed management

## Abstract

The advent of the “Green Revolution” was a great success in significantly increasing crop productivity. However, it involved high ecological costs in terms of excessive use of synthetic agrochemicals, raising concerns about agricultural sustainability. Indiscriminate use of synthetic pesticides resulted in environmental degradation, the development of pest resistance, and possible dangers to a variety of nontarget species (including plants, animals, and humans). Thus, a sustainable approach necessitates the exploration of viable ecofriendly alternatives. Plant-based biopesticides are attracting considerable attention in this context due to their target specificity, ecofriendliness, biodegradability, and safety for humans and other life forms. Among all the relevant biopesticides, plant essential oils (PEOs) or their active components are being widely explored against weeds, pests, and microorganisms. This review aims to collate the information related to the expansion and advancement in research and technology on the applications of PEOs as biopesticides. An insight into the mechanism of action of PEO-based bioherbicides, bioinsecticides, and biofungicides is also provided. With the aid of bibliometric analysis, it was found that ~75% of the documents on PEOs having biopesticidal potential were published in the last five years, with an annual growth rate of 20.51% and a citation per document of 20.91. Research on the biopesticidal properties of PEOs is receiving adequate attention from European (Italy and Spain), Asian (China, India, Iran, and Saudi Arabia), and American (Argentina, Brazil, and the United States of America) nations. Despite the increasing biopesticidal applications of PEOs and their widespread acceptance by governments, they face many challenges due to their inherent nature (lipophilicity and high volatility), production costs, and manufacturing constraints. To overcome these limitations, the incorporation of emerging innovations like the nanoencapsulation of PEOs, bioinformatics, and RNA-Seq in biopesticide development has been proposed. With these novel technological interventions, PEO-based biopesticides have the potential to be used for sustainable pest management in the future.

## 1. Introduction

According to United Nations (UN) estimates, the world’s population is rapidly growing each year and is projected to reach about 9.7 billion people by 2050 [1]. This population upsurge is coupled with a rise in the living standards of many people, and thus, there is a growing need for food supplies. However, in the climate crisis scenario that the world faces today, crop yields are consistently dropping, which is also having an impact on how efficiently and effectively food is produced [2]. To make matters worse, food security has been seriously affected by the emergence of the COVID-19 outbreak. Evidently, the pandemic has wiped out year-long improvements in agricultural productivity, and now, in the coming decade, approximately 30 million more people are anticipated to experience a hunger crisis [3]. Therefore, it becomes imperative to take immediate actions to improve crop productivity without compromising the ecological integrity of the agroecosystems. Over the years, the accelerated pace of farm mechanization in agriculture has popularized the use of intensive agronomical approaches for achieving immediate crop improvisation [4]. For instance, pesticides were largely promoted for use in agriculture around the middle of the 20th century [5]. However, despite the increased food security and other advantages that synthetic pesticides offer, they are harmful to the environment and many of its inhabitants [6]. Therefore, presently, these intensive modern agricultural methods based on the indiscriminate use of synthetic fertilizers and pesticides have come under fire [7,8,9]. Out of the total usage, about 10% of synthetic pesticides reach the target pests, whereas ~90% remain in the environment and further enter human resources via run-off [10]. Pesticide toxicity in rural farmers and their families has also been reported because of the manual spraying of chemical molecules [11]. The widespread use of these synthetic pesticides in agriculture has led to environmental contamination, pesticide residues in food, a threat to nontarget organisms, and persistence in the fields [12]. However, the reinforcement of strict laws and a comprehensive pesticide registration process has reduced the number of pesticides available on the market. Simultaneously, it has paved the way for the incorporation of natural pesticides into agricultural systems [13].

Lately, to reduce pesticide loads in soils, the focus of current research has shifted to sustainable farming, and biopesticides are being widely pitched for this purpose [14,15]. Biopesticides are substances with unique biological activities that can be utilized for agricultural pest control [16]. They can be obtained from a variety of living organisms, including plants, nematodes, bacteria, viruses, and fungi. Among these diverse groups, the use of botanicals (plant-based biopesticides) is gaining worldwide popularity in vector management to prevent the introduction of synthetic toxins into the food chain [16]. Before the widespread use of chemical pesticides (around 2000 years ago), plants were the primary and ancient sources of pest control [17]. As a result, the dictum “All the pests that out of Earth arise, the Earth itself the antidote supplies” (Lithica circa 400 B.C.) is a prescription that has inspired agricultural experts to devise popular ways of biopesticide-mediated crop protection [18]. Due to their ecofriendly characteristics, these biopesticides can be used in integrated pest management [15,19,20,21]. To increase crop output, botanical pesticides, such as crude extracts or purified or partially purified products from plants, can be applied singly or in combination with other substances against a variety of pest vectors [10]. Owing to their biodegradable nature, lack of persistence, quick decomposition, no harmful effects on groundwater, and agroprotective properties, botanical pesticides have been recognized as ecofriendly alternatives to chemical pesticides [22]. Plant products are mainly extracted using various isolation procedures, whereas plant extracts are prepared by crushing the dried plant parts and dissolving them in suitable solvents.

Among the various plant products, essential oils (hereafter referred to as plant essential oils or PEOs) are complex mixtures of volatile compounds found in aromatic plants [23]. PEOs are the first pick for many biopesticides largely because of their various bioactivities [24], such as antibacterial [25], antioxidant [26], anti-inflammatory [27], cytotoxic [28], anticancer [29], and antileishmanial [27]. Nearly 300 of the 3000 PEOs known so far have commercial applications, which ensures a demand of 3,70,000 metric tons by 2024 with an estimated market capitalization of USD 13.94 billion [30]. PEOs are synthesized in the secretory structures of plant organs as secondary metabolites (such as monoterpenes, sesquiterpenes, and phenylpropanoids) to confer direct and indirect defense against herbivores and pathogens [31]. Therefore, most of the studies nowadays are directed towards these metabolically active secondary metabolites. In addition to providing protection to plants, PEOs play a pivotal role in different ecological interactions, including allelopathy, and are beneficial to society for their utility in various perfume, food, and pharmaceutical industries [32]. However, the essential oil composition of plants can be influenced by many factors, such as plant cultivar [33], geographical area [34], extraction method [35], etc. Of late, the commercialization of many PEOs is in contemplation, and specific measures are being put forward to achieve PEOs that are less volatile and highly stable. In this regard, potential micro- and nanoencapsulation systems can be a suitable approach to improving the stability and bioavailability of PEOs [36]. At this juncture, considering the present “food stress” situation, it is crucial to amass this useful information on PEO-based biopesticides and acknowledge their commercialization for the good of the community. Therefore, the objectives of this review were: (i) to collate up-to-date information on the use of PEOs as biopesticides (bioherbicides, bioinsecticides, and biofungicides) and their mode of action using bibliometric analysis; (ii) to highlight the latest technological advancements such as nanoencapsulation, bioinformatics, and transcriptomic studies on PEO-based biopesticides; and (iii) to identify and discuss various constraints of PEOs related to their commercialization and safety aspects. The outcome of this review will help researchers gain a thorough understanding of the evolution of research on biopesticides and devise future dimensions in this area for attaining sustainability in agriculture.

## 2. Bibliometric Analysis of Research on PEOs’ Biopesticidal Potential

**Figure 1:**
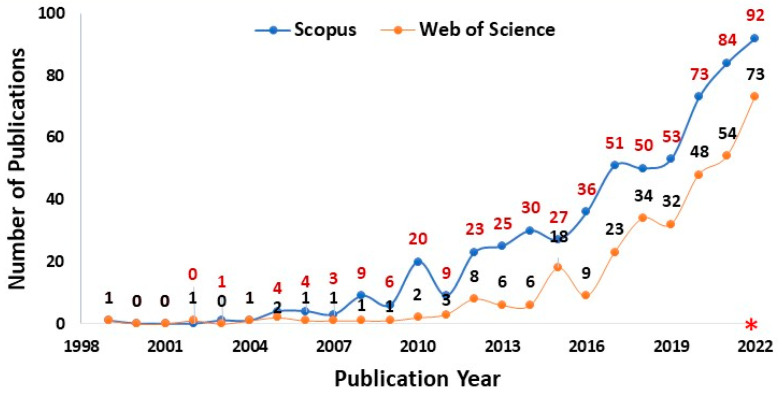
Year-wise trend in publication in the Scopus (blue dots with maroon-color numbers) and Web of Science (orange dots with black-color numbers) databases on the topic “plant essential oils as biopesticides” based on the search query: PUBYEAR > 1998 AND TITLE-ABS-KEY (“essential oils”) AND (“biopesticides” OR “bioinsecticides” OR “biofungicides” OR “bioherbicides”) (Source: [40,41]). * denotes incomplete dataset for the year 2022.

**Figure 2:**
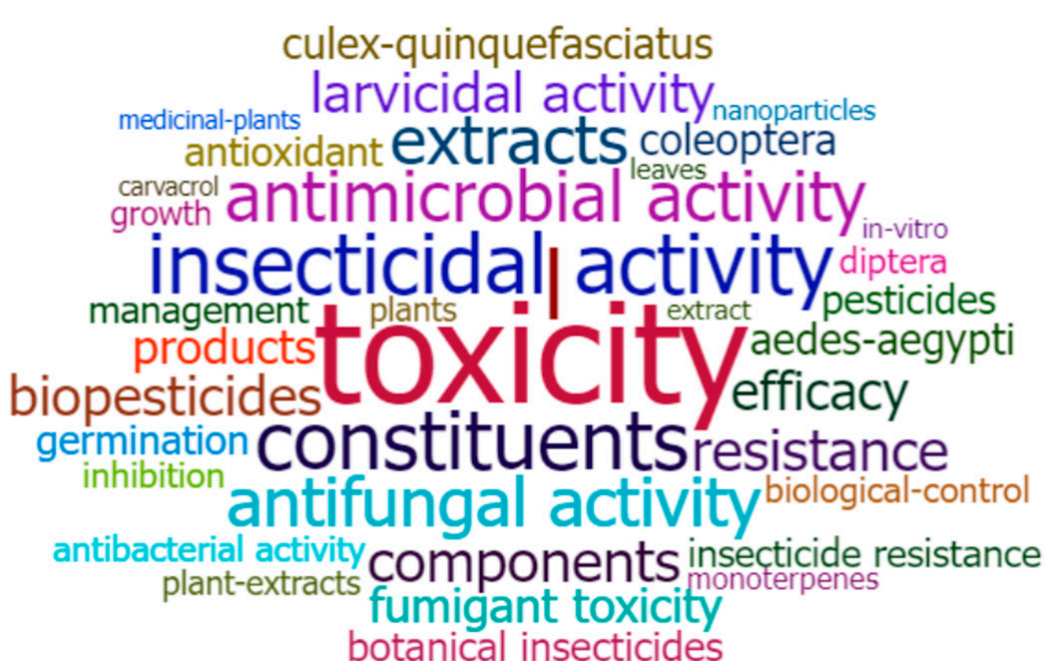
Word-cloud diagram depicting the major research highlights on the topic “plant essential oils as biopesticides”, based on the search query: [PUBYEAR > 1998 and TITLE-ABS-KEY (“essential oils”) AND (“biopesticides” OR “bioinsecticides” OR “biofungicides” OR “bioherbicides”)] (Source: [41]).

**Figure 3:**
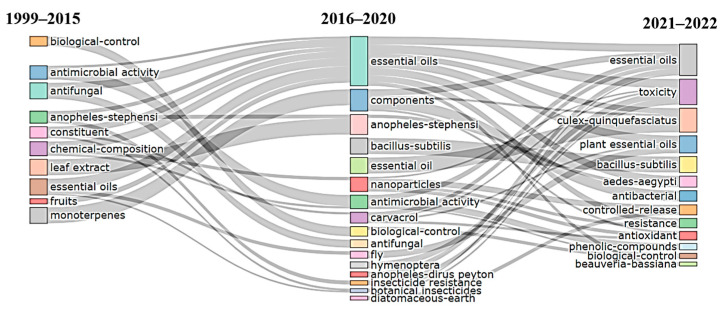
Thematic evolution plot depicting the evolution of research trends and their convergence as well as divergence under three different time intervals: (i) 1999–2015, (ii) 2016–2020, and (iii) 2021–2022, for the search query: [PUBYEAR > 1998 AND TITLE-ABS-KEY (“essential oils”) AND (“biopesticides” OR “bioinsecticides” OR “biofungicides” OR “bioherbicides”)] (Source: [41]).

**Figure 4:**
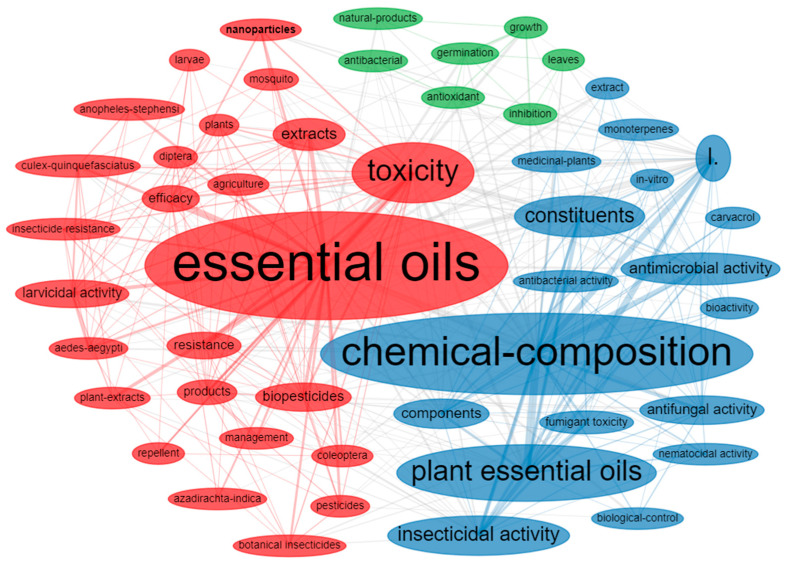
Keyword co-occurrence network plot depicting the major research themes and their interconnections on the topic “plant essential oils as biopesticides” based on the search query: [PUBYEAR > 1998 and TITLE-ABS-KEY (“essential oils”) AND (biopesticides” OR “bioinsecticides” OR “biofungicides” OR “bioherbicides”)] (Source: [41]).

Bibliometric analysis is an effective statistical technique to systematically assess and visualize the development of literature related to a particular subject or research area [12]. We conducted a bibliometric analysis on 21 December 2022, using various search queries in the Scopus and Web of Science databases to observe the trends in research on the roles of PEOs as biopesticides during the last 25 years (1999–2022). The search query was: [PUBYEAR > 1998 and TITLE-ABS-KEY (“essential oils”) AND (“biopesticides” OR “bioinsecticides” OR “biofungicides” OR “bioherbicides”)]. By limiting the search to English language, the query was further refined by selecting the publication types (articles, reviews, conference or proceeding papers, book chapters, and data papers only) published up to 2022. The search query produced a total of 328 documents from the Web of Science and 602 documents from Scopus (Table S1). For both databases, the number of publications produced on the topic per year is illustrated in Figure 1. Due to the wider and more up-to-date coverage of the literature on this topic, results obtained from the Web of Science database were considered for further analysis [12]. The bibliometric analysis was performed using the “bibliometrix” package in R (version 4.2.1) software [37,38], and the keyword-plus (ID) dataset was used for a detailed analysis [39]. Based on the results of the analysis, the global pattern of research on PEOs as biopesticides is presented in Table S2, which compiles a list of the top 10 leading researchers or authors, sources or journals, countries, and affiliations or institutions working on this topic. Pictorial representations such as a word cloud (Figure 2), a network map of keyword co-occurrences (Figure 3), and a thematic evolution plot (Figure 4) are also included in this review to give a clear picture of the research trends in this topic [12,39]. Other useful information as part of the bibliometric analysis is given in Table S1. Briefly, the search query in the Web of Science database yielded 328 documents (viz., 266 articles, 62 reviews, 4 proceeding papers, 1 book chapter, 1240 keywords plus, and 1071 authors’ keywords) from 150 sources developed by 1479 authors (Table S1). In the subsection that follows, a detailed explanation of the findings of the bibliometric analysis is provided.

### PEOs as Biopesticides: Research Trends

The findings of the search query revealed that publications on the topic: “PEOs as biopesticides” first appeared in 1999 (1 document), and that the topic began to receive more attention in 2012 (>8 documents) and afterwards (Figure 1). With an annual growth rate of 20.51%, more than 48 documents have been published in the Scopus and Web of Science databases over the past three years (Figure 1). This clearly indicates that the attention of many eminent scientists has been dragged into this topic, and there is an increasing number of studies investigating the biopesticidal potential of PEOs, particularly in the last five to ten years. Of note, Senthil-Nathan S, Benelli G, Maggi F, Vasantha-Srinivasan P, and Pavela R have been recognized as the top five researchers globally working on this topic, with the number of publications varying between 22, 19, 13, 13, and 11 documents, respectively (Table S2). Most of the top nations supporting research on this topic are mainly from the European (Italy and Spain), Asian (India, China, Iran, and Saudi Arabia), and American (Brazil, USA, and Argentina) regions (Table S2). The University of Camerino (Italy), King Saud University (Saudi Arabia), Manonmaniam Sundaranar University (India), the University of Pisa (Italy), and the Federal University of Viçosa (UFV, Brazil) top the list among the major institutions by publishing 23, 22, 21, 20, and 19 documents, respectively (Table S2). The top five journals publishing research documents in the domain of pesticides and biopesticides over the last 25 years are *Industrial Crops and Products* (26), *Plants—Basel* (17), *Molecules* (16), the *International Journal of Tropical Insect Science* (9), and *Agronomy—Basel* (8) (Table S2). The topic of our review falls under the inclusive purview of these journals, which primarily focus on publishing research on sustainable pest management and environmental safety.

Based on the Web of Science keyword-plus datasets, most of the research on PEOs in the field of pest management has focused on various domains such as toxicity, chemical components, pesticidal actions (such as insecticidal, antifungal, antimicrobial, antibacterial, etc.), extracts, efficacy, resistance development, antioxidants, and studies on insect groups (Figure 2). Further, the evolution in research on PEOs as biopesticidal agents from 1999 to 2022 was grouped into three time periods, namely, 1999–2015, 2016–2020, and 2021–2022 (Figure 3). The findings showed that the initial phase (1999–2015) of research was primarily focused on essential oil extraction, chemical composition, and utilization for biological control (such as antimicrobial and antifungal activities), using insects as model organisms and monoterpenes as a key biopesticide agent. For the time span from 2016 to 2020, convergence in some areas has been observed, and the research areas have been widened with the discovery of PEO components like carvacrol as a potential biopesticide, insecticide resistance, efficacy, the incorporation of nanotechnology in biopesticide formulations, and gene expression studies using some model organisms. The controlled release of biopesticides using immobilization techniques like nanoparticles, insect resistance to chemical pesticides, toxicity evaluation, and biological control, particularly for insects, have all received significant attention in recent years (Figure 3). This demonstrates a definite progression of research towards the sustainable management of ecosystems using biopesticides.

According to a co-occurrence network plot, three main subcategories of PEO research were identified as: (i) essential oils with a focus on their toxicity, biopesticide potential, efficacy, insecticidal activities, and product formulations incorporating nanoparticles; (ii) analysis of the chemical composition of essential oils obtained from various plants, including medicinal plants, with a focus on exploring their biocontrol potential, particularly antimicrobial (antifungal and antibacterial) and insecticidal activities, using monoterpenes and carvacrol as major PEO constituents; and (iii) the antioxidant and antibacterial properties of natural products derived from leaves of plants with a focus on germination and plant growth inhibition via allelopathy and phytotoxicity (Figure 4). The first two groups interact with several nodes to build a robust cluster. Besides the core focus of these groups on different themes, they are interrelated and provide a broad spectrum of research on the bioinsecticidal potential of PEOs. The findings of the bibliometric analysis are further elaborated in various sections of the review.

## 3. PEOs as “New Age Biopesticides”

Recently, the development of biopesticides from PEOs has been increasingly considered one of the most sought-after areas of research [42]. Various methods such as distillation (hydrodistillation, steam distillation, or dry distillation) or mechanical means are commonly used for the extraction of pure PEOs from leaves [34], roots [43], stems [44], fruits [45], seeds [33], fruit peels [46], wood [47], bark [48], resin [49], flowers [50], etc. Most of these plant parts are rich in a variety of secondary metabolites, e.g., terpenes, aldehydes, ketones, phenolics, etc. Among these groups, various subcategories may be identified as: monoterpenes (e.g., D-limonene, α-pinene, myrcene, and eucalyptol), terpene alcohols (e.g., geraniol and linalool), terpene aldehydes (e.g., citral and citronellal), ketone alcohols (e.g., carvone), phenolic terpenes (e.g., thymol and carvacrol), phenylpropanoids (e.g., eugenol), terpene oxides, and sesquiterpenes [51]. PEOs have been established as suitable products for biopesticides, but their commercial popularity is hindered due to high volatility, faster degradation, and reduced water solubility. Therefore, to overcome these limitations, PEOs are encapsulated inside different matrices (such as polymers) for the preservation of their biological properties and increase in durability [52]. On a global basis, herbicides account for the most applied pesticides, followed by insecticides and fungicides. Other groups, such as rodenticides and nematicides, constitute the minority and have a lesser number of biopesticidal studies under their names. The following subsections highlight the degree to which PEO-based biopesticides, particularly bioherbicides, bioinsecticides, and biofungicides, can be used in the agriculture sector, their modes of action, the contribution of technological advancements, as well as the challenges involved in shifting to these healthier alternatives.

### 3.1. PEOs as Bioherbicides

Driven by the growing selection pressure of synthetic herbicides, a transition away from exclusive reliance on these herbicides has gained critical importance due to the development of herbicide resistance in various weed species [53]. To reduce the use of these synthetic herbicides that degrade the environment, the PEOs and their constituents can be useful in sustainable weed management [28,54,55,56,57,58]. The weedicide properties of PEOs are in fact governed by allelopathic interactions (chemically mediated plant–plant interactions) and allelochemicals (chemicals responsible for allelopathic effects) [59]. Benvenuti et al. [60] found that aqueous PEOs (at 10, 100, and 1000 mg L^−1^) extracted from various Asteraceae family members, particularly the *Artemisia* species and *Xanthium strumarium* L., negatively affected the germination of weeds (*Amaranthus retroflexus* L. and *Setaria viridis* [L.] P. Beauv.). The main active constituents identified in these PEOs were artemisia ketone, 1,8-cineole, *trans*-non-creole, chrysanthenone, *β*-pinene, camphor, borneol, yomogi alcohol, and artemisia alcohol [60]. The aqueous PEO of *Citrus aurantiifolia* (Christm.) Swingle and its major constituents (citral and limonene) were found to exhibit phytotoxicity (at 0.1–1.5 mg mL^−1^ EO) and cytotoxicity (at 0.01–0.10 mg mL^−1^ EO) in tested weeds [28]. In a recent study by Gruľová et al. [58], the PEO of *Juniperus horizontalis* Moench (water–acetone mixture; at 1, 5, 10, and 50 µg mL^−1^) showed a detrimental effect on the germination of weeds, particularly *Trifolium pratense* L. and *Lolium perenne* L. Sabinene, a major component of *J. horizontalis* PEO, also displayed antigerminative activity (at 0.5, 5, and 100 µg mL^−1^) as well as a phytotoxic effect on the root length of *L. perenne* at all the concentrations [58]. Many researchers have documented the herbicidal effects of PEOs from different *Eucalyptus* species. For example, PEOs (water–acetone mixtures) of *Eucalyptus gunnii* Hook.fil. and *E. pulverulenta* Link (at 100, 250, 500, and 1000 mg mL^−1^) were phytotoxic to the weed, *Portulaca oleracea* L. [61]. 1,8-cineole was identified as the major active component in both PEOs, inter alia, α-pinene in the former and β-pinene in the latter [61]. PEOs (water–acetone mixture) of *Eucalyptus bicostata* Maiden, Blakely and Simmonds, *E. gigantea* Hook.fil., *E. intertexta* R.T.Baker, *E. obliqua* L’Hér., *E. pauciflora* Sieber ex Spreng., *E. tereticornis* Sm., *E. cinerea* F.Muell., and *E. nicholii* Maiden and Blakely, at concentrations ranging from 100, 250, 500, and 1000 µg mL^−1^, decreased the radicle elongation and germination of weeds, *Sinapis alba* L., *S. arvensis* L., and *Lolium multiflorum* Lam. [62,63]. Eucalyptol (or 1,8-cineole) was identified as the principal component in these oils [62,63]. Commercially available PEOs of *Eucalyptus citriodora* Hook., *Lavandula angustifolia* Mill., and *Pinus sylvestris* L. (at 0.125–1.0 µL mL^−1^) were found to be effective at controlling weeds, while having little or no impact on crop plants [54]. Linalool, 1,8-cineole, and camphor were reported as the dominant constituents [54]. Other reports on aqueous PEOs of *Pinus radiata* D. Don. and *Cupressus sempervirens* L. (at 1.0–6.0 µL mL^−1^ in Petri dishes) have reported the phytotoxic effect of these oils on the germination and growth of several weeds, including *S. arvensis*, *Lolium rigidum* Gaud, *Trifolium campestre* Schreb, and *Phalaris canariensis* L. [64,65]. *α*-pinene, *α*-cedrol, *δ*-3-carene, germacrene D, *β*-pinene, and limonene were identified as the major EO components in these studies [64,65]. Similarly, PEOs (dimethyl sulfoxide (DMSO)–water solution) of various *Origanum* species (*Origanum syriacum* L., *O. onites* L., and *O. majorana* L.) (at 5, 10, and 20 µL Petri^−1^) decreased the rate of germination of various weeds, including *Thlaspi arvense* L., *A. retroflexus*, *Rumex crispus* L., and *Lactuca serriola* L., in Petri plate assays and greenhouse experiments [66]. Carvacrol, thymol, and *α*-terpineol were majorly present in these oils [66]. From the above studies, the herbicidal effects of different PEOs or their constituents are readily apparent, which means attempts to replace the use of synthetic herbicides with natural plant products can be tried on a large scale. In contrast to chemical pesticides with roughly 20 modes of action, natural products can deploy multiple mechanisms against weeds to support their function as highly effective bioherbicides with reduced resistance [15,20,67]. For instance, PEOs can decrease seed germination and plant growth by inhibiting the activity of α-amylases [61]. Previously, synthetic herbicides were reported to function in weed management by attacking the photosynthetic apparatus of target plants. The adoption of a similar mechanism by PEOs is highly likely [68].

#### Effect of PEO Supplementation on the Photosynthesis and Ultrastructure Integrity of Weeds

**Figure 5:**
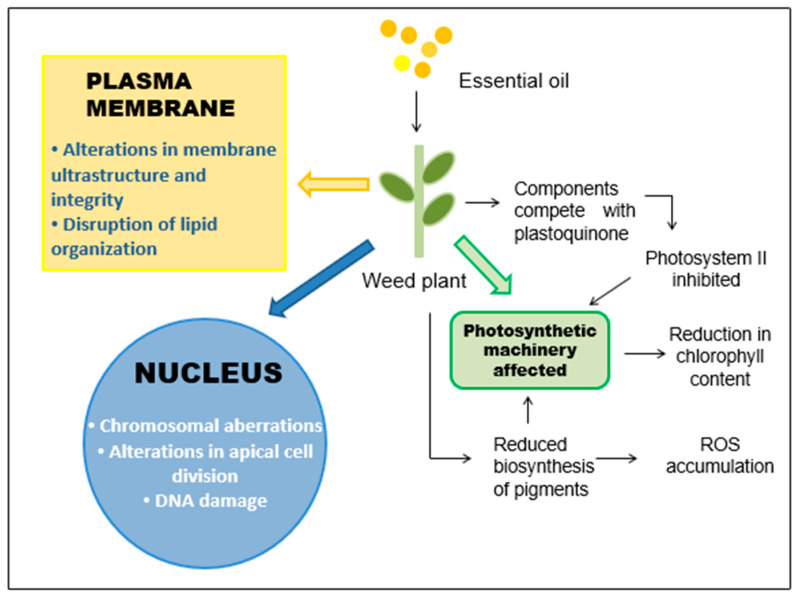
Effect of plant essential oils on the photosynthetic apparatus and ultrastructure integrity of weeds. Abbreviations: ROS: reactive oxygen species.

Chlorophyll content is a reliable indicator of photosynthetic efficiency, and thus, a drop in this value is indicative of the negative effect of PEOs on the photosynthetic machinery of weeds [69] (Figure 5). For example, the PEO of *Artemisia fragrans* Willd. reduced the biosynthesis of photosynthetic pigments, decreased photosynthesis, and inhibited the electron transport chain in a perennial weed, *Convolvulus arvensis* L. [70]. “Sarmentine”, an important herbicide obtained from *Piper* sp. PEO, has been reported to inhibit photosystem II (PS II) activity by competing with the binding sites for plastoquinone (an electron transporter involved in photosynthesis). Other detrimental effects of this herbicide, such as a disruption of plasma membrane integrity, were also noticed in target weeds [71]. At the ultrastructural level, the integrity of weed plants’ organelles is complicated by shocks such as induction of chromosomal aberrations by PEOs (Figure 5). A report by Issa et al. [72] clearly observed that the supplementation of *Vitex negundo* L. PEO affected apical cell division and induced DNA damage in the root tips of *Allium cepa* L. Thus, it can be said that the mechanism of action of PEOs on weeds varies from plant to plant and is largely determined by the chemical constitution of various PEOs or their constituents. For example, phenylpropanoids may operate via binding to membrane receptors, whereas monoterpenes may disrupt the lipid organization [73].

### 3.2. PEOs as Bioinsecticides

The use of plants or parts of them as insecticides has been widely documented in the past. However, it is only lately that PEO demand has increased in sustainable pest management due to their toxicity on various crop pests [74]. Therefore, to ascertain the efficacy of PEOs and encourage the shift to these alternatives, the insecticidal potential of PEOs has been compared with the synthetic pesticides commercially available in the market. For instance, spraying aqueous PEO of *Foeniculum vulgare* Mill. (at 1.2, 2.5, 5.0, 10.0, and 15.0 mL L^−1^) was highly effective in inducing the mortality of a polyphagous aphid, *Myzus persicae* (Theobald), using the soil organisms *Harmonia axyridis* (Pallas) and *Eisenia fetida* (Savigny) as positive controls. The active constituents of the oil were *trans*-anethole and fenchone [75]. By contrast, aphid mortality using a commercial pesticide, Vaztak (α-cypermethrin), was coupled with harmful effects on nontarget species, unlike PEO [75]. Using the same positive controls, the bioinsecticidal potential of PEO spray (diluted in DMSO/acetone or water for different insects) from *Schizogyne sericea* (L.fil.) DC. was confirmed against four insect pests at the following EO concentrations: 1.2, 2.5, 5.0, 10.0, and 15.0 mL L^−1^ [76]. PEO of *Piper betle* L. (at 0.5, 1.0, and 1.5 mg L^−1^) was reported to exhibit larvicidal activity against *Aedes aegypti* L., and the wild strains were susceptible to oil but resistant to a 0.1 mg L^−1^ dose of insecticide (Temephos) [77]. With LD_50_ and LD_90_ values of 860 and 1300 µg insect^−1^, respectively, the PEO of *J. horizontalis* (dissolved in acetone; at 435.0, 652.5, 870.0, and 1740.0 µg insect^−1^) demonstrated larvicidal action against *Tenebrio molitor* L. (yellow mealworm) [58]. Promising adulticidal, larvicidal, and ovicidal properties of PEO from *Citrus grandis* (L.) Osbeck (dissolved in DMSO or acetone; at 10–1000 mg L^−1^) have also been reported by Sarma et al. [78]. Likewise, PEOs of *Cuminum cyminum* L., *Mentha longifolia* L., and *Allium sativum* L. were insecticidal to the crop pests *Sitophilus granarius* L. and *Rhyzopertha dominica* (F.) [79]. The effect was, however, maximal with *A. sativum* PEO (at 15 and 20 µL Petri^−1^) for the former pest and (10, 15, and 20 µL Petri^−1^) for the latter [79]. According to Uştüner et al. [80], the PEO of *Eucalyptus camaldulensis* Dehnh. (at 10 and 20 µL Petri^−1^) was found to be insecticidal against a variety of storage pests, including *R. dominica*, *S. granarius*, *Tribolium confusum* du Val., *Callosobruchus maculatus* (Fabricius), and *Acanthoscelides obtectus* (Say). Particularly at the higher dose (20 µL), PEOs of *Ocimum basilicum* L., *Rosmarinus officinalis* L., and *Artemisia dracunculus* L. induced mortality in the adults of *T. confusum* and *A. obtectus* [81]. The toxicity of volatile monoterpenes (dissolved in ethanol) and aromatic compounds (at 10 and 20 µL Petri^−1^) naturally present in different PEOs has also been reported against *S. granarius* adults [82]. Additionally, most studies have described the involvement of different PEOs in the elimination of storage pests such as weevils, beetles, house flies, blowflies, and aphids [83,84,85,86,87,88]. Other reports on the nematicidal effects of different PEOs against plant-parasitic nematodes are also available [89,90]. Besides these individual studies on PEOs and their insecticidal potential, recent investigations have shifted the pace towards the formulation of insecticides that combine both natural (PEOs or their components) and synthetic analogues to overcome insecticide resistance and accelerate their lethal potential. For example, the potency of menthol (at 0.1 and 1.0 µM) has been confirmed with a carbamate insecticide, Bendiocarb [91]. Several PEOs have also been reported to enhance the toxicity of Permethrin insecticide [92].

#### Current Status and Outlook for the Identification of Receptor Modulators That Enhance PEOs’ Insecticidal Effects

**Figure 6:**
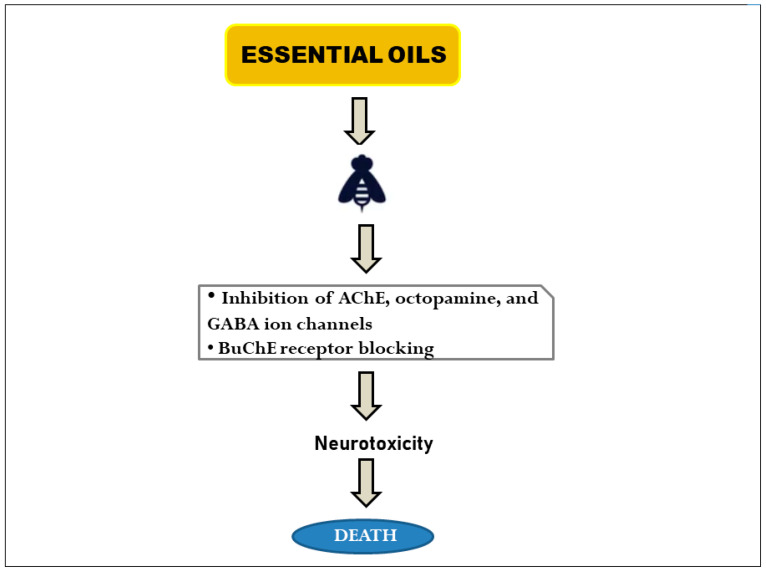
Inhibition of insect-specific receptors aids in the bioinsecticidal activity of plant essential oils. Abbreviations: GABA: gamma-aminobutyric acid; AChE: acetylcholinesterase; BuChE: butyrylcholinesterase.

Considerable progress has been made over the years in figuring out which PEOs are toxic to insects. According to several studies, PEOs have insecticidal [93,94], insect-repellent [48,95,96], antifeedant [97,98], and insect-growth-regulatory activities [99,100]. As such, researchers all over the world are viewing PEO use as the best means to protect crops against pests and trying to comprehend their mechanism of toxicity on a variety of insect species. For instance, monoterpenes (major constituents of many PEOs) show a cytotoxic effect on insect tissues by decreasing cell membrane permeability, reducing the number of intact Golgi bodies and mitochondria, and impairing cellular respiration. Since the nervous system is crucial to the functional integrity of insects, most PEOs find it to be an easy target of action [101]. Numerous putative receptors for PEO activity have been described in the literature, some of which include receptors for acetylcholinesterase (AChE), octopamine, adenosine triphosphatases (ATPases), gamma-aminobutyric acid (GABA)-gated chloride channels, butyrylcholinesterase (BuChE), and nicotinic acetylcholine [102,103,104]. AChE is a crucial enzyme involved in the breakdown of the neurotransmitter acetylcholine into choline and acetate, the termination of neurotransmission, and synaptic signaling [105]. Previously, AChE activity has been found to be inhibited by PEOs of *Cyclotrichium niveum* (Boiss.) Manden and Scheng, *Thymus praecox* subsp. *caucasicus* (Willd. ex Ronniger) Jalas var. *caucasicus*, and *Anethum graveolens* L. [106,107,108]. Additionally, monoterpenes in PEOs can inhibit AChE and cause neurotoxicity in living organisms, particularly arthropods (Figure 6). According to Re et al. [109], monoterpenoids like linalool act upon an insect’s nervous system and affect the ion transport and release of the AChE enzyme. Therefore, a suppression of AChE activity is a biomarker of PEO-induced toxicity in insects [105,110].

Besides acetylcholine, octopamine is another essential neurotransmitter, neuromodulator, and neurohormone found in abundance in the insect nervous system. PEOs can modify neuron activity through octopamine receptors and lead to a complete breakdown of the insect nervous system [111]. Octopamine receptors are G-protein–coupled receptors, and their role in mediating the acaricidal activity of PEOs has been pointed out in the literature [92,105]. Tyramine is another neuroactive substance found in insects that has similar properties to octopamine. Neuromodulatory effects may result from the interactions between insect tyramine receptors and monoterpenes due to an increase in intracellular calcium (Ca^2+^) and a decrease in cAMP (cyclic adenosine monophosphate) levels [112,113]. The ionotropic GABA receptors, which control how the neurotransmitter GABA signals in insects, are among the other targets of PEOs. These receptors are normally involved in the inhibition of neurotransmission; however, PEOs can act as receptor antagonists and potentiate or inhibit the insect nervous system [114]. Al-Harbi et al. [115] compared the insecticidal efficacy of PEOs from lavender (*L. angustifolia*), black seeds (*Nigella sativa* L.), and basil (*O. basilicum*) against *Sitophilus oryzae* L., one of the main storage pests. Lavender and basil PEOs up-regulated the expression of genes associated with the detoxification system of *S. oryzae*, including *DCL1294*, *CL8*, and a *CYP450* gene (*CYP4Q4*), thereby indicating the biopesticidal effectiveness of these oils [115].

### 3.3. PEOs as Biofungicides

Today, there is evidence that the use of some chemical fungicides causes long-term, severe, negative effects on human health and the environment, and therefore, primary reliance on these synthetic pesticides for managing plant diseases is highly undesirable. In this context, PEO-based biofungicides can be regarded as environmentally benign alternatives that can prevent plant diseases, particularly those caused by plant-pathogenic fungi [116]. Many in vitro studies on PEOs have been conducted so far to expand the knowledge base on PEOs’ mucocidal activity [50,117]. For this, fungi are either procured from pathology centers or isolated from various contaminated sites, such as infected plant parts and heritage objects [50,117]. A number of publications on PEOs have reported their crucial roles in minimizing the commercial losses of fruits caused by postharvest diseases [118,119,120,121]. For instance, several PEOs (at 500–1000 µL L^−1^) could prevent citrus fruit decay caused by the fungus *Geotrichum citri-aurantii* (Ferraris) E.E. Butler [118]. Here, the PEO droplets were fungistatic and inhibited the growth of fungus, but they were unable to completely eradicate the fungal colony [118]. By contrast, other researchers have discovered that PEOs are more effective against fungi in the vapor phase [120,121]. Studies have reported the effect of *Thymus vulgaris* L. and *Cinnamomum verum* J.Presl PEOs (vapor phase; at 16.7 µL L^−1^) in managing the brown rot in peaches caused by *Monilinia laxa* (Aderh. and Ruhland) Honey [122] and that of *C. sempervirens* EO (at 25, 50, 100, 200, 400, and 1000 µg mL^−1^) in managing the grey mold in tomatoes caused by *Botrytis cinerea* Pers. [123]. The principal constituents (viz., *α*-pinene, *α*-cedrol, and *β*-caryophyllene) in the *C. sempervirens* oil displayed maximum antifungal activity [123].

Gruľová et al. [124] demonstrated that the PEO of *Origanum vulgare* O.F.Müll exhibited strong antifungal activity against a variety of plant-pathogenic fungi, including *Monilinia fructicola* (G. Winter) Honey, *Aspergillus niger* van Tieghem, *Penicillium expansum* Link, and *B. cinerea*, particularly at 500 and 1000 mg L^−1^ concentrations of the oil. This was explained by the lipophilic nature of phenolic compounds (carvacrol and thymol) present in oil, which kill fungal cells by interfering with enzymes involved in protein synthesis, energy production, and membrane stability [125]. Nine species of saprophytic fungi (*Penicillium* sp.) that primarily infect citrus fruits were successfully inhibited from growing by PEOs (in vapor phase or incorporated in growth medium) of *Satureja* species (*Satureja cuneifolia* Ten., *S. cilicica* P.H. Davis, *S. montana* L., *S. hortensis* L., *S. thymbra* L., and *S. spicigera* (K. Koch) Boiss.) at 10, 20, and 30 µL concentrations of the oil [126]. Likewise, PEO of *E. camaldulensis* (dissolved in DMSO–water solution; at 0.25, 0.5, and 1.0 mg mL^−1^) inhibited the growth of fungal mycelium in *Verticillium dahliae* Kleb, *Fusarium oxysporum* Schl., *Phytium debaryanum* Auct. non R. Hesse, and *Sclerotinia sclerotiorum* (Lib.) de Barry, within seven days of oil application [80]. PEOs of *Eucalyptus cinerea* F.Muell. and *Eucalyptus nicholii* Maiden and Blakely (diluted in ethanol; at 10 µL concentration) were effective against two plant-pathogenic fungi, *B. cinerea* and *F. oxysporum* f.sp. *lycopersici* [63]. Antifungal activities of PEOs (4 µL mL^−1^) from *Pinus pinea* L. and *Pinus nigra* J.F. Arnold have been reported against *B. cinerea*, *Bipolaris sorokiniana* (Saccardo) Shoemaker, *Fusarium culmorum* (W.G.Smith) Saccardo, *Fusarium graminearum* Schwabe, and *Fusarium avenaceum* (Corda) Saccardo [127]. Limonene, *α*-pinene, and *β*-pinene were reported as the major active constituents in these oils. The PEO of *C. sempervirens* also inhibited the growth of a variety of plant-pathogenic fungi [64]. Recently, Parikh et al. [128] carried out a Petri plate assay and determined a strong inhibition of mycelial growth and spore germination by 38 PEOs (at 0.125, 0.25, 0.5, 1.0, 2.0, and 4.0 mL L^−1^) in fungal pathogens of common pulses, including *Aphanomyces euteiches* Drechsler, *B. cinerea*, *Colletotrichum lentis* Damm, *Didymella pisi* Chilvers, Rogers and Peever, *Didymella rabiei* (Kovachevski) Arx, *Didymella lentis* W.J. Kaiser, B.C. Wang and J.D. Rogers, *F. avenaceum*, *Stemphylium beticola* Woudenberg and Hanse, *Sclerotinia* sp., and *Pythium* sp.

#### Mechanism of Action of PEOs in Fungal Cells

**Figure 7:**
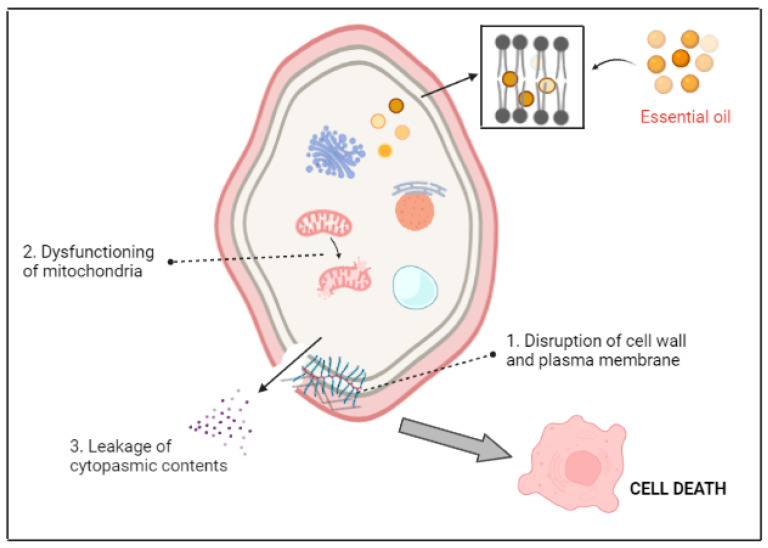
Mechanism of action of plant essential oils as potential biofungicides in fungal cells. PEO entry results in disruption of the plasma membrane, cell wall, and important cell organelles, which causes efflux of intracellular constituents and fungal cell death.

Many promising studies relating to the role of PEOs as efficient fungal toxins have been put forward. Taken together, the studies have described morphological and structural alterations in the fungal mycelium, viz., disruption of the cell wall and membrane, cytoplasmic coagulation, and damage to plant organelles, as possible effects of PEOs [129,130] (Figure 7). For example, clove oil was reported to be fungitoxic as it inhibited the growth of a saprophytic pathogen, *Colletotrichum gloeosporioides* (Penzig) Penzig and Saccardo, in sweet cherry plants [131]. Evidently, the morphology and structural integrity of *C. gloeosporioides* were found to be completely distorted by clove oil application under scanning electron microscopy (SEM) and transmission electron microscopy (TEM). Some of the effects observed were a broken fungal cell wall and endomembrane system, leaky cell membranes, and the release of intracellular constituents outside of the fungal cell [131]. Additionally, PEOs may damage important cell organelles and result in twisted and flattened fungal hyphae with debris exposed on the surface [132,133]. PEOs have a strong affinity for ergosterol (a sterol specifically found in fungi), which ultimately results in the increased permeability of the cell membrane, the inhibition of ATPase-mediated hydrogen (H^+^) ion efflux, cellular swelling, hyphae breakage, and fungal cell death [134,135].

The literature has provided conclusive evidence that PEOs are lipophilic in nature, a fascinating property that allows them to permeate the target cell wall and cytoplasmic membrane easily. However, this deprives the target cell of its protective layers of fatty acids, polysaccharides, and phospholipids, thereby increasing its permeability [136]. In general, the path of PEO-induced toxicity flows from alterations in membrane permeability and an imbalance in intracellular osmotic pressure to distorted plant organelles and leakage of cytoplasmic contents and ATP molecules (Figure 7). Previously, the PEO of *Mentha cardiaca* L. has been reported to promote ion leakage, particularly Ca^2+^, potassium (K^+^), and magnesium (Mg^2+^) ions in *Aspergillus flavus* Link from LHP-PV-1 cell membranes [137]. Ultrastructural alterations in *Phytophthora infestans* (Mont.) de Bary, a late blight disease-causing fungal pathogen, were observed upon exposure to different PEOs [138]. In another study, *A. niger* treated with a mixture of PEOs (extracted from *Thymus eriocalyx* (Ronniger) Jalas and *Thymus x-porlock*) showed severe mitochondrial rupture and damage to the fungal cell wall and plasma membrane [139]. PEO of *Ageratum conyzoides* L. induced various structural changes in *A. flavus* fungal cells, like decreased ridge polarization in the mitochondrial cristae and the formation of notably rough and invaginated vesicles of the plasma membrane [140]. Cinnamon PEO resulted in shrunken conidia, disrupted hyphae, and atypical cell masses in *A. flavus* [141]. Tao et al. [142] also noticed changes in the ultrastructure of *Penicillium italicum* Wehmer, particularly in the plasmalemma and cytoplasm, upon treatment with citral (a PEO constituent).

Overall, PEO-based biopesticides have shown immense potential for regulating the growth and development of several weeds, pests, and microbes by inhibiting different cellular and molecular mechanisms. The application methods, composition of essential oils, and their concentrations play a major role in the efficient control of the pests. However, to further improve the efficacy of the PEO-based biopesticides, a detailed understanding of the mode of action at the molecular level and related technological advances needs to be explored. The next section of the review sheds some light on this aspect.

## 4. Technological Advancements in the Research on Biopesticides

### 4.1. Use of Nanoformulations

Since the role of botanicals (including those based on PEOs) as biopesticides has been well-established in the literature, the commencement of a new era involves newer technologies being explored, researched, and implemented. As of late, significant progress has been made in the development of novel formulations for plant-based biopesticides. With the disappearance of conventional formulations, which included liquid formulations (e.g., emulsions and suspensions) or dry formulations (e.g., dust, powders, granules, wettable powders, and water-dispersible granules), this increases the attractiveness of substituting newer technologies with biopesticides [143,144,145]. Newer biopesticides with improved stability, shelf life, and consumer safety are highly beneficial due to their competitive advantage over conventional counterparts [145]. Beyond the above sections, which extensively describe the bioactivities of PEOs, their industrial usage is limited due to constraints such as low solubility, low bioavailability, and high volatility. Therefore, to improve PEO bioactivity and stability, many researchers have suggested the encapsulation of PEOs in chemical matrices that act as carriers and facilitate the controlled release of oils. For the development of stable emulsions, nano- and microformulations based on the encapsulation of a plant’s active components onto a matrix appear to be highly promising [52,146].

Nanosized emulsions, being kinetically stable, are superior to microsized ones due to their low volatility, long-lasting stability, and requirement of a lower surfactant concentration, unlike microemulsions. Moreover, they have negligible flocculation and allow for the controlled release of active compounds that possess bioherbicidal, biofungicidal, and bioinsecticidal properties [147,148] (Table 1). Since PEOs are insoluble in water, they require large quantities of toxic solvents for their dissolution. To overcome this problem, studies advocating the use of solvent-free methods for the nanoencapsulation of PEOs are proving to be significant milestones in research [149]. Thus far, a wide range of polymeric carriers have been developed for the encapsulation of PEOs, which offer multiple benefits, most importantly the controlled release of PEOs during storage or application [150]. Encapsulated particles or composites can be prepared through emulsification, coacervation (with gelatin or gum arabic), spray drying (with maltodextrin), complexation (with cyclodextrin), ionic gelation (with chitosan), nanoprecipitation (with poly DL-lactide-co-glycolide, i.e., PLGA), and film hydration [151]. Lopes et al. [152] reported protected release and better cytotoxicity in plant extracts encapsulated with chitosan or lipid-based carriers against Sf9 insect cell lines in comparison to commercially available pesticides. Likewise, the antifungal activity of PEO from “Palmarosa” encapsulated in nanostructured lipid carriers has been reported against *Aspergillus nomius* Kurtzman, B.W. Horn, and Hesselt in both in vitro (mycelia growth inhibition) and in situ (against precontaminated Brazil nuts) set-ups [153]. Nanoemulsions of *S. hortensis* PEO were herbicidal on the selected weeds and caused membrane disruption and physiological malfunction in these plants [154]. The size and stability of nanoemulsions are largely determined by the source of the plant. For instance, nanoemulsions of PEOs from *Artemisia* sp. remained stable even after 28 weeks of storage when a 3:1 concentration of oil and surfactant was used [155].

Table 1 provides useful insights into the research relating to the encapsulation of PEOs in different carriers along with their mechanism of action on target cells. Regarding PEO-based biopesticides, novel formulations formed by mixing the active principles of oil with other compounds are being popularized. For instance, pure “azadirachtin” alone was less effective than the one formed in a neem oil medium [156]. Additionally, the biopesticidal properties of nanoformulations made from both pure PEOs and their constituents are quite impressive [149].

**Table 1:** List of plant essential oil (PEO)-based nanoemulsions/nanoparticles used in formulation of biopesticides (viz., bioherbicides, bioinsecticides, and biofungicides) along with their effects on various biological targets. Abbreviations: LC_50_: the lethal concentration which causes the death of 50% of test animals; RC_50_: the effective concentration value for 50% repellency.

Source of PEO or Its Active Component	Encapsulation/ Emulsifying Agent	Method(s) Used for Encapsulation	Biological Target	Effects	Reference(s)
Bioherbicides					
*Satureja hortensis* L.	Carbohydrate and protein natural polymers (gum arabic /gelatin, apple pectin, and gelatin) and cross-linkers (citric acid and transglutaminase enzyme)	Complex coacervation	*Amaranthus retroflexus* L. and *Solanum lycopersicum* L.	(a) Inhibition in germination and growth of *A. retroflexus* (b) Inhibition was comparable to chemical herbicide (Metribuzin)	[157]
*Foeniculum vulgare* Mill.	Tween 80	Ultrasonic emulsification	*Phalaris minor* Retz., *Avena ludoviciana* Durieu, *Rumex dentatus* L., and *Medicago denticulata* Willd	(a) Stability of nanoemulsions persistent after 30 days of storage (b) Inhibition of target weeds at as low as 0.05 wt.% dose of nanoemulsion (c) Individual components of PEO showed weed growth at high doses	[158]
*Carum carvi* L. and *Mentha piperita* L.	Commercial multifunctional adjuvant ATPOLAN BIO 80 EC	Emulsification	*Echinochloa crus-galli* (L.) P. Beauv.	(a) Foliar injuries were reported and photosynthetic efficiency was reduced in weed plant at 2.5% adjuvant dose (b) Crop plant was unaffected	[159]
Bioinsecticides					
*Cymbopogon nardus* (L.) Rendle	Tween 80	Emulsification	*Oryzaephilus surinamensis* L.	(a) Nanoemulsions displayed greater adult mortality than pure oil in both male and female adults (b) LC_50_ value of nanoemulsions was significantly less than that of pure oil depicting 50% mortality at lower concentrations of nanoemulsions	[160]
*Pimpinella anisum* L., *Foeniculum vulgare* Mill., and *Mentha piperita* L.	Tween 80	Self-emulsification	*Bactrocera oleae* Gmelin	(a) No residual toxicity reported (b) All nanoformulations were capable of reducing oviposition punctures (c) *P. anisum* displayed the highest percentage of repellent activity followed by *F. vulgare* and *M. piperita*	[161]
*Rosmarinus officinalis* L., *Lavandula angustifolia* Mill., *Mentha piperita* L.	Lipid: Softisan Surfactants: Kolliphor RH40 and Labrafil	Phase inversion temperature (PIT) method	*Aphis gossypii* (synonym of *Aphis forbesi* weed), *Spodoptera littoralis* Boisduval, and *Tuta absoluta* Meyrick	(a) Bioassay confirmed high mortality in *A. gossypii* treated with oil-loaded lipid carriers (b) Reduction in progeny was observed. With no mortality observed, the feeding activity of *S. littoralis* was reduced with *L. angustifolia* and *R. officinalis* carriers (c) No effect was observed on *T. absoluta*	[162]
*Foeniculum vulgare* Mill., *Mentha piperita* L., and *Citrus sinensis* (L.) Osbeck	5% Tween	Spontaneous emulsification	*Rhyzopetha dominica* Fabricius	(a) All the PEOs displayed repellent activity against the tested storage pest. *F. vulgare* oil displayed the least activity (b) Insects recovered after 24 h of treatment and habituation was validated	[52]
*Baccharis reticularia* DC. and oil components (limonene, *α*-pinene, and *β*-pinene)	Nonionic surfactant (mixture of sorbitan monooleate, polysorbate 80, and/or polysorbate 20)	Low-energy titration	*Tribolium castaneum* Herbst	(a) Both the PEO and its components displayed good repellent activity at 8.8 μg cm^−2^	[149]
*Citrus sinensis* (L.) Osbeck	Silica (SiO_2_)	Sol–gel microencapsulation	*Spodoptera littoralis* Boisduval and *Aphis gossypii* (synonym of *Aphis forbesi* Weed)	(a) Insecticidal activity against cotton leafworm (*S. littoralis*) (b) Reduction in the fertility and number of *A. gossypii* offsprings	[163]
*Pimpinella anisum* L., *Artemisia vulgaris* L., *Foenicum vulgare* Mill., *Allium sativum* L., *Lavandula angustifolia* Mill., *Mentha piperita* L., *Rosmarinus officinalis* L., and *Salvia officinalis* Pall.	5% Tween 80	Spontaneous emulsification	*Tribolium confusum* Duval	(a) All PEOs displayed repellent activity against the tested storage pest (b) With a RC_50_ value of 0.033 mg, the PEO of *P. anisum* exhibited maximum repellent activity	[164]
*Cymbopogon citratus* (DC.) Stapf and *Eucalyptus globulus* Labill.	Polysorbate 80	High-energy emulsification	*Musca domestica* L. and *Lucilia cuprina* Wiedemann	(a) No adulticidal activity with *E. globulus* oil (b) Free *C. citratus* oil displayed better adulticial activity as compared to nanoemulsion; however, the use of nanoemulsion is suitable as it prevents the volatilization and degradation of oil (c) The concentration of nanoemulsion, time of treatment exposure, and encapsulation method play significant roles in mediating adulticidal activity	[165]
*Lippia multiflora* Mold.	89.75% Hydrolate and 0.25% Chitosan	Low-energy emulsification	*Plutella xylostella* L., *Brevicoryne brassicae* L., *Hellula undalis* Fabricius, *Spodoptera exigua* Hubner, and *Bemisia tabaci* Gennadius	(a) PEO nanoemulsions were tested against synthetic pesticide, i.e., Karate 5 EC (Lambda cyhalothrin 52 gL^−1^) in two regions of Ivory Coast (b) Treatment with nanoemulsion reduced the damage of cabbage head as compared to synthetic pesticide, thereby signifying better protection against selected insects	[166]
Biofungicides					
*Cymbopogon martini* (Roxb.) W.Watson	Lipids (cocoa butter and sesame oil)	Melt emulsification method	*Aspergillus nomius* Kurtzman, B.W.Horn and Hesselt.	(a) 100% inhibition of *A. nomius* was displayed by nanostructured lipid carriers	[153]
*d*-Limonene	Emulsifiers	Phase transition composition emulsification	*Pyricularia oryzae* Cavara, *Rhizoctonia solani* J.G.Kühn, *Colletortrichum gloeosporiodes* (Penz.) Penz. and Sacc., and *Phomopsis amygdali* (Delacr.) J.J.Tuset and M.T.Portilla.	(a) Stability of nanoemulsions was tested and found to increase with an increase in dose of emulsifier (b) Emulsifier (EL40) displayed the highest stability © Inhibition of growth of tested fungal pathogens was reported	[167]
*Cinnamomum verum* J.Presl, *Thymus vulgaris* L., and *Melaleuca alternifolia* (Maiden and Betche) Cheel	Crodamol GTCC and polysorbate 80	Phase inversion composition method (PIC) and ultrasonication	*Fusarium culmorum* (Wm.G.Sm.) Sacc., *Phytophthora cactorum* (Lebert and Cohn) J.Schröt. *Trichophyton mentagrophytes* (C.P.Robin) Sabour., *Microsporum gypseum* (E.Bodin) Guiart and Grigoraki, *Aspergillus niger* Tiegh., and *Scopulariopsis brevicaulis* (Sacc.) Brainier	(a) Fungicidal activities of pure oil, macroemulsions (PIC-based), and nanoemulsions (ultrasonication-based) were reported (b) Nanoemulsions prepared using high-energy ultrasonication showed better fungicidal activities (c) Among the different PEOs studied, *M. alternifolia* oil displayed the best activity	[168]

### 4.2. Use of Bioinformatics

Studies on the importance of using PEOs as biopesticides are now moving at a rapid pace thanks to advances in bioinformatics. Bioinformatics tools can map the interactions of PEOs or their active constituents with their biological targets and treat multifactorial diseases, indicating the wide applications of this field in pharmacology [169]. Lately, by using different computational tools or in silico methodologies, many researchers have made significant contributions to this field. For example, Loza-Mejía et al. [170] conducted an in silico study involving docking-based virtual screening to determine the insecticidal potential of active compounds from the genus *Calceolaria* against insect target proteins. Likewise, the interaction of a principal compound from the wood extracts of *Tabebuia heptaphylla* with the target, i.e., ILE125 amino acid from *A. aegypti* odorant binding protein (AaegOBP1 receptor), confirmed the repellent activity of the extracts against *A. aegypti* [171]. The ability of active compounds from “Negramina” PEO to kill aphids was validated through their interaction with the insect transient receptor potential (TRP) channels [172], whereas the therapeutic potential of peppermint (*M. piperita*) against grey mold (*B. cinerea*) was confirmed by docking studies [173]. In another docking study, the role of insect tyramine receptors in mediating the responses of PEOs against insects was described [174]. Contrary to these studies, Sierra et al. [175] reported the detoxification response of mosquitoes against PEO constituents by molecular docking. The detoxification of a toxic xenobiotic component, *p-cymene*, by chemosensory proteins (CSP) present in *A. aegypti* larvae confirmed the role of CSPs in hampering the natural larvicidal activity of *E. camaldulensis* PEO [176]. Thus, the emergence of in silico studies constitutes perhaps the most significant research on biopesticides because they require fewer resources to hypothesize future physiological research and give researchers the option of conducting multiple trials to determine the appropriate interactions.

### 4.3. Transcriptomic Profiling to Elucidate PEOs’ Insecticidal Efficacy

Insect transcriptome profiling has become one of the most utilized approaches in recent years for examining the molecular basis of the insecticidal activity of numerous PEOs [176,177]. Several molecular targets of interest for plant pathologies have been discovered through expression studies using RNA sequencing. This novel technology has completely revolutionized transcriptome analysis, allowing the quantification of gene expression and the identification of new genes at an unprecedented pace.

A comparative transcriptome analysis of *Sitophilus zeamais* (Mochul’skii) in response to the PEO of *Melaleuca linariifolia* var. *alternifolia* Maiden and Betche revealed that 3562 differentially expressed genes (DEGs) were found to be involved in insecticide detoxification and mitochondrial function. Of these DEGs, 2836 genes were up-regulated, whereas 726 genes were down-regulated [176]. The mapping of DEGs identified an increase in the expression of several genes, including cytochrome P450s (*CYP450*), glutathione *S*-transferases (*GSTs*), carboxylesterases, ATP-binding cassette transporters (ABC transporters), and those associated with respiration and metabolism of xenobiotics [176]. However, PEO treatment reduced the expression of genes encoding enzymes involved in respiration, electron flow, and energy synthesis in the mitochondria. Another study by Liao et al. [178] reported that fumigation with the PEO of *M. alternifolia* induced the expression of 2208 DEGs, and the NAD^+^/NADH (nicotinamide dehydrogenase) enzyme was identified as the prime target of action for oil in insects. The oil resulted in abnormal structural changes and severe damage to the insect mitochondria and directly blocked electron transport through the mitochondrial respiratory pathway, ultimately leading to insect death. Similarly, the effect of *M. alternifolia* PEO on the morphology and ultrastructure of mitochondria in *B. cinerea* has been reported to cause mitochondrial dysfunction by reducing the activities of important mitochondrial enzymes and those involved in the tricarboxylic acid (TCA) cycle [179]. The fumigation of terpinen-4-ol (a component of *M. alternifolia* PEO) in *S. zeamais* induced the up- or down-regulation of roughly 592 DEGs in insect RNA [180]. Here, several DEGs encoding detoxification enzymes were identified, including 16 *CYP450s*, 14 esterases (*ESTs*), 10 UDP-glucuronosyltransferases (*UGTs*), 8 *GSTs*, and 2 ABC transporter genes. Of particular interest was the consistent overexpression of the genes encoding *P450s*, *GSTs*, and *ESTs* after terpinen-4-ol exposure, which sheds light on the crucial role of these genes in the systemic metabolic responses of insects [180].

Muturi et al. [181] assessed the larvicidal activity of PEO from *Commiphora erythraea* (Ehrenb.) Engl. and its fractions against three mosquito species, namely, *Culex restuans* Theobald, *C. pipiens* L., and *A. aegypti*. Real-time PCR (polymerase chain reaction) analysis revealed that the expression of *CYP450s* (*CYP6M11* and *CYP6N12*) and a *GST* gene (*GST-2*) involved in xenobiotic detoxification by mosquito larvae was significantly up-regulated by PEO treatment. The GST enzyme is also a potential molecular target of PEO from *Cymbopogon citratus* (DC.) Stapf in Asian long-horned ticks, *Haemaphysalis longicornis* Neumann, which induces the enzyme at a significantly higher rate following exposure to sublethal concentrations of the oil [182]. However, there is a paucity of information on the molecular mechanism underlying GST enzyme action, which, if available, may open new avenues for the development of effective pest management techniques for ticks and tick-borne diseases [182]. More recently, with the advent of RNA-Seq as the technology of choice for gene expression analysis, a set of genes associated with adrenergic signaling/Ca^2+^ channels, apoptosis, focal adhesion, cGMP-PKG (cyclic guanosine monophosphate-dependent protein kinase G) signaling, ECM (extracellular matrix)–receptor interaction, ubiquitin-mediated proteolysis, the mTOR (mammalian target of rapamycin) signaling pathway, and the longevity regulating pathway were identified in ticks exposed to *C. citratus* PEO and its constituent, citronellal [183]. Up-regulation of most of the genes involved in Ca^2+^ signaling (*CACNAID*, *ADCY9*, *TPM1*, and *MYH6*) and apoptosis (*CYC*, *DRONC*, *CASP7*, *CASP9*, *BCL2L1*, and *BCL-xL*) by PEO treatment was found to induce neurotoxicity and cytotoxicity in insects. Further, the toxicity of oil on insects was expected to be a product of complex factors, including oxidative stress due to increased ROS accumulation, reduced ATP levels, mitochondrial depolarization, increased intramitochondrial free Ca^2+^, and either necrotic or apoptotic death induction in ticks [183]. The activation of *CYP450* genes by several terpenoids found in PEOs has also been predicted by RNA-Seq [176,184].

Two potentially relevant genes are *cathepsin* and *lipase*, both of which play significant roles in larval development and reproduction. The importance of these genes is evident in a study by Hegedus et al. [185], which reported that the down-regulation of *CatB/CatB*-like and *CatL/CatL*-precursor in crucifer root maggot (*Delia radicum* L.) and RNAi-mediated silencing of *CatL*-precursor in red flour beetle (*Tribolium castaneum* Herbst) negatively affected mid-gut metamorphosis, tissue remolding, and larval fat body decomposition in the former, while causing 100% mortality in the latter. Further, through a complete transcriptome analysis of *T. castaneum*, Gao et al. [177] reported that the PEO of *Artemisia vulgaris* L. exerted its insecticidal activity by increasing the expression of genes encoding antioxidant enzymes, copper-zinc-superoxide dismutases, heme-peroxidases, and various transcription factors in beetles. From these studies, many gene-specific responses pertaining to PEO treatment have been highlighted in insect species. But, to discover novel, efficient, and environmentally friendly alternatives for controlling insect pests, researchers in the future will have to focus on more transcriptomic studies to investigate the molecular targets of various PEOs in insects.

## 5. Efficiency of PEOs in Promoting Sustainable Agriculture

Presently, the control of crop diseases caused by pests, weeds, etc. mainly relies on massive doses of chemical pesticides, whose extensive use has affected diverse ecosystems and mankind [186]. Potential threats from these pesticides include environmental pollution, harmful impacts on nontarget organisms, and reduced global food security [12]. Undoubtedly, the integration of food security with sustainable agroindustrial value chains and the adoption of environmentally benign biopesticides are impressive achievements in this direction [8,187]. Sustainable agriculture involves lower production and environmental costs in a system that generates higher net returns [8,39]. With this view in mind, the interests of many agricultural scientists and biotechnologists have been dragged into nanotechnology to encourage the use of biological nanopesticides encapsulated in different nanocarriers [9]. As noted in the bibliometric analysis and discussed under previous headings, the nanoencapsulation of biopesticides has proven to be an efficient approach for the conservation and protection of PEOs from outside degradation. PEO nanoemulsions can facilitate the efficient delivery of pesticides to target sites. Therefore, the incorporation of PEOs as biopesticides has many useful applications in agronomy, with the most explicit one being an ecofriendly and cost-effective method of pest and weed management by PEOs. Besides pest control, a multitude of PEO benefits in the agriculture and food industries have been reported [186,188]. More importantly, the use of PEO nanobiofertilizers can cut down on the amounts of chemical fertilizers used in agriculture fields, improve soil and crop quality, and increase the nutrient use efficiency of crops. The food industry also utilizes PEO biofilms for antimicrobial packaging and food preservation [186].

Recently, numerous attempts have been made to develop methods and find various ways to utilize waste produced post-PEO extraction for nutritive purposes [189]. These waste products are rich in secondary metabolites and can secure the supply chain. A supply chain gives information about the availability, intrinsic costs, and storage conditions of the raw material and provides a clear understanding of the feasibility analysis and production process. Cid-Pérez et al. [190] suggested the presence of antioxidant and antimicrobial properties in solid waste residues of PEOs, though the activities were lesser in comparison to the oil. In brief, from the above discussion, the use of PEOs as biofertilizers and biopesticides is supposed to be a fundamentally sustainable outlook for modern-day agriculture. However, the widespread commercialization of plant-based biopesticides is hampered by certain constraints related to their use, as described in the following section.

## 6. Constraints, Safety, and Commercialization Considerations

### 6.1. Constraints

As the science of biopesticides gets rooted in agronomical practices, certain loopholes need to be addressed to achieve economically suitable products. The following are the major drawbacks of using natural botanical pesticides in sustainable agricultural practices:

- The increase in quantity and frequency of PEO application is a result of the degradation and volatilization of essential metabolites upon exposure to air, sunlight, moisture, and high temperatures. As was previously stated, the nanoencapsulation of PEOs can practically serve this purpose if used at the appropriate concentration. For example, Su et al. [191] developed several derivatives based on PEOs and β-methoxyacrylate to improve the stability of fungicidal oil formulations.
- Due to technological barriers, these natural compounds are less economical and not as popular among farmers as synthetic pesticides. However, this problem can be overcome by providing subsidies to farmers and highlighting the long-term benefits of botanical pesticides by various governmental and nongovernmental agencies.
- In contrast to chemical pesticides that are usually lethal, biopesticides often function by preventing the pathogens from proliferating further. Thus, combined spraying of chemical and biological pesticides is being adopted to get rid of pre-existing pests. However, to achieve a complete phase-out of chemical pesticides, more research needs to be conducted to explore the broad spectrum of lethal natural products as biopesticides.
- The requirements of a large arable land area, source plants, and proper climatic conditions are additional barriers to the use of biopesticides. The collection of plant material needs to be conducted at the right time to preserve the quality of the raw materials required, thus making it seasonal. The purity of products is also restricted due to the differences in chemical composition of plants’ active ingredients in different geographical areas [192].

### 6.2. Safety

In addition to the above-mentioned constraints, certain safety measures need to be recognized before the application of PEO-based biopesticides in fields. Some of these are listed below:

- Harmful organic solvents used for PEO solubilization, as well as toxic doses acquired by nonpesticidal active components of plant products, raise concerns about the safety of plant-based biopesticides [193].
- Modifications in the physical and chemical processes of the soil ecosystem can result from interactions between PEOs and soil microbes [146,194].
- The growing body of research on nanoemulsions raises alarms about the safety of such formulations, partly due to the involvement of organic solvents in their lab-scale preparation and the use of spray drying on a commercial scale, which is potentially hazardous to the environment.

Overall, if appropriate agricultural legislation is put forward to drive the commercialization of botanical biopesticides, farmers’ extensive knowledge of plant products can be leveraged [195]. As such, many nations are establishing policies and regulations to promote the use of biopesticides for sustainable agriculture [17,196]. In fact, due to their natural pesticidal properties, the US government has exempted the use of plant extracts and PEOs from registration [197].

### 6.3. Commercialization

Much academic research is being conducted on biopesticides; however, they are met with certain drawbacks, due to which their industrial implementation is lagging [197]. Moreover, the large-scale commercialization of plant-based biopesticides is questionable due to a dearth of studies that evaluate their efficacy in real-world settings [198]. Of late, only a few studies have brought to light the commercial applications of various PEOs or their active components (Table 2). For instance, Borges et al. [171] speculated on the incorporation of plant wood extracts into gels and lotions for commercial purposes after detecting the repellent activity of active components present in these extracts. Tangtrakulwanich and Reddy [199] argued that a blend of active compounds in plant-based biopesticides reduces the occurrence of resistance in pests, although complete resistance is unavoidable due to ongoing natural selection. Thus, such studies are paving the way for active research that considers real-world scenarios and large-scale field environments outside of the controlled laboratory environment. Recently, the utilization of PEOs in the cosmetics and pharmaceutical industries has already been elucidated by many researchers [200,201,202].

In agricultural setups, however, the commercial use of such natural products is limited to pocket-unfriendly organic farming, which is one of the main reasons for their decreased popularity. The commercialization of PEO-based products is further hindered due to various laws and IPRs (intellectual property rights). In European markets, PEO-based products are being used for fungicidal and insecticidal purposes, while those exhibiting herbicidal action are still scarce. By contrast, several PEO-based herbicidal formulations have seen commercial success in the USA [203]. According to a survey (between 1997 and 2010) by Cantrell et al. [204] on the registration status of 277 new active ingredients (NAIs), conventional synthetic pesticides accounted for most registrations (78%), while 54.8% of NAIs were recorded for natural biopesticides. This suggests that the commercialization of natural plant-based biopesticides has been gaining attention for a long time and has become a major driver of sustainable agriculture. Overall, the multiple modes of action of botanical pesticides pose a great challenge for pests in achieving resistance against them, making them a perfect choice for commercialization.

## 7. Conclusions

The “back to nature” approach has seen a rise in the usage of plant-based biopesticides over synthetic analogues, which have been labelled as potentially hazardous to the environment and human health. The use of PEOs or their active constituents as biopesticides makes them highly sought-after in the field of ecological agriculture. PEOs work in line with synthetic pesticides, yet they can be differentiated based on their unique properties like easy biodegradability, higher structural diversity, novel molecular targets, and little or no mammalian toxicity. These properties help them achieve sustainable crop yields. Even though PEOs serve as alternatives to chemical pesticides, there are many challenges, such as volatility and disparity in bioefficacy under laboratory and field conditions, that hinder their commercialization. Therefore, novel approaches such as the nanoencapsulation of PEOs, which enhance their bioavailability, stability, and pesticidal efficacy with reduced volatility, can be recommended for integrated pest management. However, before the widespread acceptance of newer technologies, it is pertinent to assess the viability of such products from a commercial and economic perspective. On that note, PEOs have a bright future, and the scientific community should work towards spreading awareness regarding the sustainable use of plant-based biopesticides among the masses.

## Figures and Tables

**Table 2:** List of commercially available biopesticides based on plant essential oils (PEOs) and their active components.

Type of Biopesticide	Country	PEO/Active Component	Name of Product
Biofungicide	Europe	Clove oil	BIOXEDA
Biofungicide + Bioinsecticide	Europe	Sweet orange oil	LIMOCIDE
Biofungicide + Bioinsecticide	Europe	Sweet orange oil	OROCIDE
Biofungicide + Bioinsecticide	Europe	Sweet orange oil	PREV-AM
Bioherbicide	USA	*d*-Limonene	GreenMatch
Bioherbicide	USA	Clove oil	Matratec
Bioherbicide	USA	45% Clove oil + 45% cinnamon oil	WeedZap
Bioherbicide	USA	Lemongrass oil	GreenMatch EX
Bioherbicide	USA	*d*-Limonene	Avenger Weed Killer
Bioherbicide	USA	Eugenol	Weed Slayer

## Data Availability

Not applicable.

## Supplementary Materials

The following supporting information can be downloaded at: https://www.mdpi.com/article/10.3390/plants12162916/s1. Table S1: Main information table showing bibliometric analysis details (Source: Scopus [40]; Web of Science [41]); Table S2: A list of leading (top 10) authors, sources (journals), countries, and affiliations/universities/institutions exploring the role of plant essential oils as biopesticides (Source: [41]).
