# Supplementary material for: Plant Essential Oils as Biopesticides: Applications, Mechanisms, Innovations, and Constraints
Source: Plants (Basel). 2023 Aug 10;12(16):2916. doi: 10.3390/plants12162916 (PMC10458566; doi:10.3390/plants12162916)
Supplement: Supplementary file 1 [file plants-12-02916-s001.zip › plants-2539255-supplementary.pdf]

Supporting Information

Table S1: Main information table showing bibliometric analysis details (Scopus [40]; Web of Science [41])

**Description**	**Results**	
**MAIN INFORMATION ABOUT DATA**		
	**Web of Science**	**Scopus**
Timespan	1999:2022	1999:2022
Sources (Journals, Books, etc)	150	260
Documents	328	602
Annual Growth Rate %	20.51	21.73
Document Average Age	3.29	4.52
Average citations per doc	20.91	24.53
References	18347	35955
DOCUMENT CONTENTS		
Keywords Plus (ID)	1240	3638
Author's Keywords (DE)	1071	1695
AUTHORS		
Authors	1479	2454
Authors of single-authored docs	8	28
AUTHORS COLLABORATION		
Single-authored docs	11	32
Co-Authors per Doc	5.84	5.23
International co-authorships %	35.67	27.41
DOCUMENT TYPES		
article	260	477
article; early access	2	0
article; proceedings paper	4	15
review	60	77
review; book chapter	1	30
review; early access	1	0
data papers	0	3

Table S2. A list of leading (top 10) authors, sources (journals), countries, and affiliations/ universities/ institutions exploring the role of plant essential oils as biopesticides (Source: [41]).

Authors/Sources	No. of Articles	Countries/Institutions	No. of Articles
**Top 10 authors**		**Top 10 countries**	
Senthil-Nathan S	22	Brazil	137
Benelli G	19	Italy	126
Maggi F	13	India	94
Vasantha-Srinivasan P	13	Spain	67
Pavela R	11	The United States of America (USA)	57
Thanigaivel A	11	Argentina	50
Ponsankar A	10	Egypt	46
Karthi S	9	China	37
Canale A	8	Iran	37
Campolo O	7	Saudi Arabia	37
**Top 10 journals/sources**		**Top 10 affiliations/ institutions**	
Industrial Crops and Products	26	University of Camerino, Italy	23
Plants-Basel	17	King Saud University, Saudi Arabia	22
Molecules	16	Manonmaniam Sundaranar University, India	21
International Journal of Tropical Insect Science	9	The University of Pisa, Italy	20
Agronomy-Basel	8	The Federal University of Viçosa (UFV), Brazil	17
Ecotoxicology And Environmental Safety	8	Kasetsart University, Thailand	14
Environmental Science and Pollution Research	8	National University of Córdoba, Argentina	14
Crop Protection	7	Alexandria University, Egypt	13
Frontiers in Plant Science	7	The Federal University of Sergipe (UFS), Brazil	13
Insects	7	The University of Liège, Belgium	12
